# Atrophy patterns in hippocampal subregions and their relationship with cognitive function in fibromyalgia patients with mild cognitive impairment

**DOI:** 10.3389/fnins.2024.1380121

**Published:** 2024-05-23

**Authors:** Yingming Long, Xinyan Xie, Yingwei Wang, Jinping Xu, Ziyi Gao, Xiaokun Fang, Tong Xu, Nan Zhang, Dongling Lv, Ting Wu

**Affiliations:** ^1^Department of Radiology, Affiliated Hospital of Nanjing University of Chinese Medicine, Nanjing, China; ^2^Shenzhen Institutes of Advanced Technology, Shenzhen, China; ^3^Department of Cardiology, Affiliated Hospital of Nanjing University of Chinese Medicine, Nanjing, China

**Keywords:** fibromyalgia, cognition, hippocapmus subfields, mild cognitive impairment, FreeSurfer

## Abstract

**Objectives:**

Fibromyalgia (FM) has been associated with decreased hippocampal volume; however, the atrophy patterns of hippocampal subregions have not yet been identified. We therefore aimed to evaluate the volumes of hippocampal subregions in FM patients with mild cognitive impairment (MCI), and to explore the relationship between different subregional alterations and cognitive function.

**Methods:**

The study included 35 FM patients (21 with MCI and 14 without MCI) and 35 healthy subjects. All subjects performed the Montreal Cognitive Assessment (MoCA) to assess cognitive function. FreeSurfer V.7.3.2 was used to calculate hippocampal subregion volumes. We then compared hippocampal subregion volumes between the groups, and analyzed the relationship between hippocampal subregion volume and cognitive function using a partial correlation analysis method.

**Results:**

Compared with the healthy subjects, FM patients with MCI had smaller hippocampal volumes in the left and right CA1 head, Molecular layer head, GC-DG head, and CA4 head, and in the left Presubiculum head. Poorer executive function, naming ability, and attention were associated with left CA1 head and left Molecular layer head atrophy. By contrast, hippocampal subregion volumes in the FM patients without MCI were slightly larger than or similar to those in the healthy subjects, and were not significantly correlated with cognitive function.

**Conclusion:**

Smaller volumes of left CA1 head and left Molecular layer head were associated with poorer executive function, naming ability, and attention in FM patients with MCI. However, these results were not observed in the FM patients without MCI. These findings suggest that the hippocampal subregions of FM patients might present compensatory mechanisms before cognitive decline occurs.

## Introduction

Fibromyalgia (FM) is a chronic, widespread disease that presents as musculoskeletal pain. It is characterized by diffuse abnormal nociceptive perception in the painful areas, and is accompanied by other symptoms such as fatigue, insomnia, anxiety, depression, and cognitive dysfunction. These symptoms can affect the quality of life and make it difficult for patients to engage in everyday activities (Bair and Krebs, 2020; Sarzi-Puttini et al., 2020). Globally, the average estimated prevalence of FM is between 2 and 4% (Queiroz, 2013). FM mainly occurs in females (Marschall et al., 2011) and has a misdiagnosis rate of approximately 87%. Lee et al. (2018) proposed that the main mechanism of FM is the abnormal amplification of pain signals at the central level (i.e., central sensitization). To date, much evidence indicates that central sensitization is likely to be the main pathophysiological mechanism of FM. However, peripheral sensory, emotional, and cognitive mechanisms may also be involved (Rehm et al., 2021).

Approximately 50 to 80% of FM patients reportedly experience cognitive decline (Can et al., 2012). FM patients are often diagnosed with cognitive dysfunction, which mainly manifests as memory and attention issues (Katz et al., 2004). The reported subjective cognitive difficulties in FM patients are sometimes referred to as “fibro fog.” (Walitt et al., 2016) Standardized neuropsychological scales are typically used to objectively assess cognitive function. Studies using neuropsychological scales have indicated that patients with FM have relatively poor long-term memory, attention, and executive function compared with healthy controls (HCs) (Glass, 2009; Segura-Jiménez et al., 2015). However, the mechanisms by which cognitive problems arise in FM patients are not yet clear. Several investigations into the relationship between chronic pain and cognitive impairment have suggested that pain can impair cognitive function (Dick et al., 2002; Seminowicz et al., 2004). Chronic pain leads to changes in the anatomical structures and functions of neural circuits, thereby altering cognitive function and emotion. For example, patients with chronic low back pain may have altered anatomical structures in regions associated with the pain modulation of cognitive function and mood, including the dorsolateral and medial prefrontal cortices, anterior cingulate gyrus, and insula (Seminowicz et al., 2011). Additionally, Martinsen et al. (2014) used the Stroop Color and Word Test and functional magnetic resonance imaging (MRI) to evaluate cognitive performance and brain activation patterns in FM patients. Their results indicate that the cognitive difficulties of the FM patients are mainly related to reduced activation of the caudate nucleus and hippocampus. We therefore speculate that FM patients with cognitive impairment may exhibit specific anatomical alterations of the brain.

The hippocampus forms part of the limbic region of the brain, and is renowned for its role in learning and memory. It also participates in regulating pain, cognition, and stress responses (Liu and Chen, 2009; Lucassen et al., 2014). In a recent study of more than 300,000 subjects, Zhao et al. (2023) noted that hippocampal atrophy is more severe in patients with chronic pain in multiple areas, and cognitive decline is faster. They further revealed a correlation between cognitive decline and accelerated hippocampal aging in patients, and reported that a partial decline in fluid intelligence in these patients is mediated by hippocampal atrophy. These findings suggest that the hippocampus plays an important role in the development of cognitive impairment in patients with chronic pain. Moreover, hippocampal alterations are commonly reported in FM patients. For example, it has been observed using single voxel proton magnetic resonance spectroscopy that N-acetylaspartate levels are decreased in the bilateral hippocampus of FM patients, which manifests as the metabolic dysfunction of hippocampal neurons (Emad et al., 2008). There is thus an urgent need to investigate the microscopic changes that occur in the hippocampus of FM patients, to better understand cognitive difficulties in this patient population.

A previous study compared total hippocampal volumes between FM patients and HCs, and reported that total hippocampal volume was smaller in FM patients than in HCs (McCrae et al., 2015). Anatomically, however, the hippocampus is not a monolithic body, but rather consists of multiple subregions. Although these subregions are interconnected, they have diverse morphologies and functions, and are relatively independent. An investigation of hippocampal subfield volumes may thus better reflect subtle alterations in atrophy patterns than an evaluation of total hippocampal volume, thereby supplying more valuable information in the early stages of FM (Mueller et al., 2017). The hippocampal subregions—especially the cornu ammonis (CA)1, CA3, and dentate gyrus (DG)—play vital roles in many processes, including memory, spatial navigation, and executive function (Yassa et al., 2010; Mueller et al., 2011; Tamnes et al., 2014; Suthana et al., 2015). Furthermore, the DG can mediate memory processes associated with spatial information (Kesner, 2018). It is thus essential to explore the structural changes that occur in the hippocampal subregions of FM patients. Leon-Llamas et al. (2021) reported that most hippocampal subregions are markedly reduced in female FM patients, including the hippocampal tail, Molecular layer (ML), CA3, CA4, Subiculum, Presubiculum, granule cell layer of the DG (GC-DG), and Parasubiculum. However, the specific relationships between cognitive deficits and hippocampal subregions were not identified. To the best of our knowledge, there is currently a lack of research into the structural alterations of hippocampal subregions in FM patients, and of their relationship with cognitive function.

We therefore aimed to explore hippocampal subregion volumes in FM patients with MCI, and to evaluate the relationship between selected subregions and cognitive function. We hypothesized that FM patients with MCI would exhibit various patterns of hippocampal atrophy, and that distinct hippocampal atrophy patterns would mediate cognitive function in different cognitive domains. The present study is important for our overall understanding of FM-related pathophysiological variations in the occurrence and development of cognitive impairment.

## Materials and method

### Participants

The current study was approved by the Research Ethics Committee of the Affiliated Hospital of Nanjing University of Chinese Medicine (2021NL-193-02). All participants understood the study protocol and signed a written informed consent form. We enrolled 35 HCs and 35 FM patients who attended the Affiliated Hospital of Nanjing University of Chinese Medicine from January 2022 to March 2023. The FM patients were diagnosed by two rheumatologists using the 2016 criteria of the American College of Rheumatology (Wolfe et al., 2016). The inclusion criteria for the FM group were as follows: (1) generalized pain was present, defined as pain in at least four of five regions; (2) symptoms had been present for at least 3 months; and (3) patients had a Widespread Pain Index score ≥ 7 and a Symptom Severity Scale score ≥ 5, or a Widespread Pain Index of 4–6 and a Symptom Severity Scale score ≥ 9. All enrolled subjects underwent cranial MRI.

We used the Fibromyalgia Impact Questionnaire, Hamilton Anxiety Rating Scale (HAMA), Hamilton Depression Rating Scale (HAMD), Pittsburgh Sleep Quality Index (PSQI), Montreal Cognitive Assessment (MoCA), and visual analog scale (VAS) to evaluate disease severity, neurocognitive function, and psychological impairment in all subjects. Of these, the Fibromyalgia Impact Questionnaire was completed by a rheumatologist, and the remaining evaluations were conducted by a neurologist in the Psychological Scale Unit of the Department of Neurology. In the HAMA and HAMD, total scores ≤7 were considered normal (Julian, 2011).

The MoCA is widely used as a rapid screening tool to identify mild cognitive impairment (Nasreddine et al., 2005; Berg et al., 2018). Moreover, for individuals with fewer than 12 years of education, an additional 1 point was added to the total score to correct for the impact of educational differences, as recommended by Nasreddine (Nasreddine et al., 2005). The MoCA scale tests abilities such as executive function, naming, attention, language, abstraction, delayed recall, and orientation (Nasreddine, 2021). Nasreddine et al. (2005) proposed a threshold value of 26 for the MoCA scale. In the present study, the FM patients were therefore categorized into two subgroups: those with MCI (MoCA score < 26; *n* = 21) and those without MCI (MoCA score ≥ 26; *n* = 14).

### Image acquisition

We used a Verio 3.0 T (Siemens, Munich, Germany) superconducting MRI scanner with an eight-channel phased-array head coil to acquire data. To reduce artifacts caused by head movement, a ring of foam padding was placed around the head coil. The important sequence was the sagittal three-dimensional T1-weighted image, which was acquired using the following parameters: repetition time = 2,300 ms, echo time = 2.19 ms, flip angle = 9°, matrix = 245 × 256 mm, slice thickness = 1 mm, sagittal slices = 176, slice gap = 0.5 mm, and scanning time = 7 min 16 s.

### Image processing

**Figure 1:**
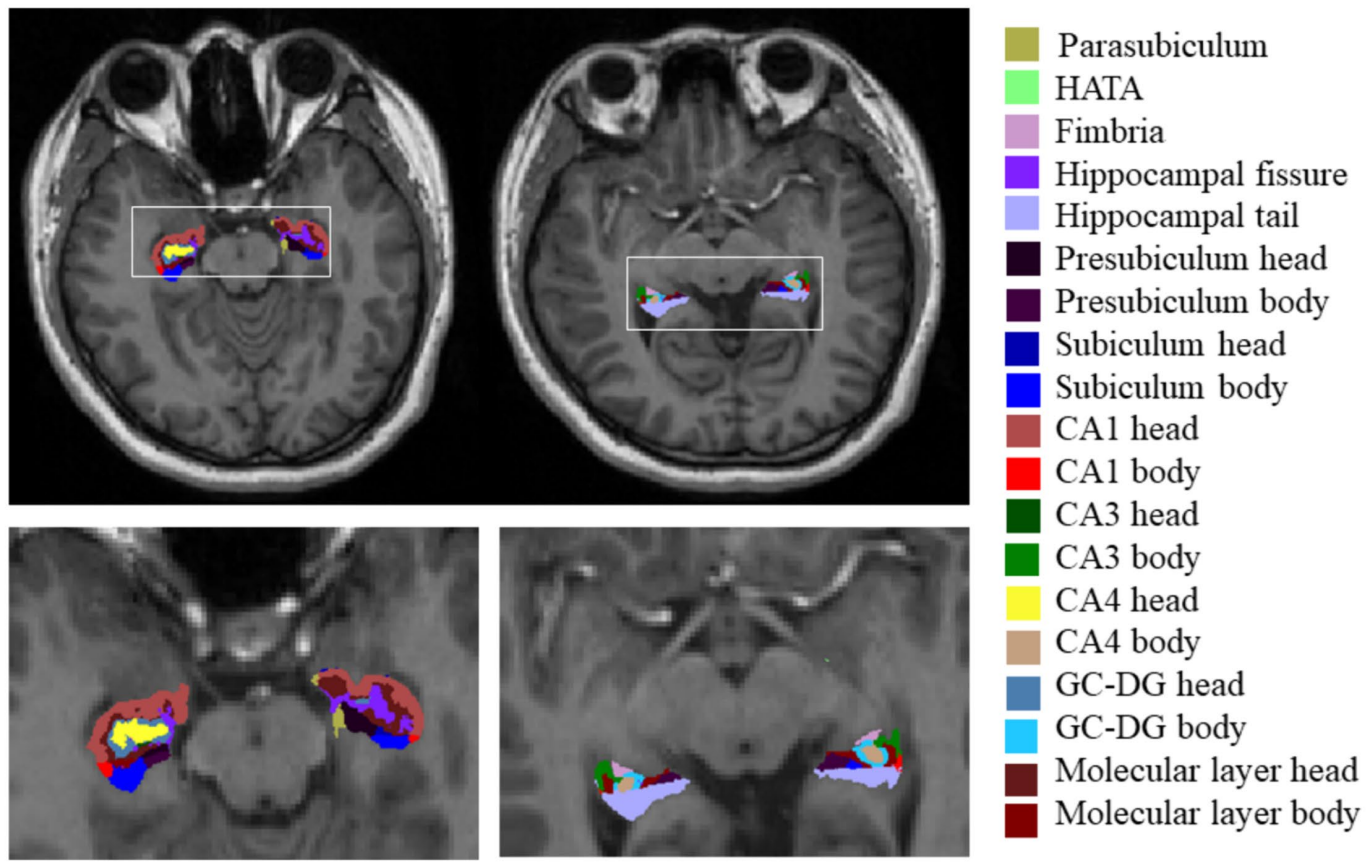
Hippocampal subfield segmentation in FreeSurfer. Axial views of hippocampal subregion anatomical maps of a fibromyalgia patient with mild cognitive impairment. CA, cornu ammonis; GC-DG, granule cell layer of the dentate gyrus; HATA, hippocampal amygdala transition area.

For the T1-weighted images, we used FreeSurfer V.7.3.2,^1^ which has an automatic subcortical segmentation function. Probabilistic mapping constructed from high-resolution MRI data was used by this tool to generate an automated segmentation of the hippocampal substructures, including the nuclei of the amygdala (Saygin et al., 2017). Joint segmentation of the hippocampus and amygdala ensured that structures did not overlap and there were no gaps between them. The protocol automatically segmented the total volume of each hippocampal hemisphere into that of 19 substructures. We then used the Freeview tool to visualize these segmented substructures (Figure 1): the Hippocampal tail, Subiculum body, CA1 body, Subiculum head, Hippocampal fissure, Presubiculum head, CA1 head, Presubiculum body, Parasubiculum, Molecular layer head, Molecular layer body, GC-DG head, CA3 body, GC-DG body, CA4 head, CA4 body, Fimbria, CA3 head, and HATA (hippocampal amygdala transition area). We also extracted the estimated total intracranial volume (eTIV) from the segmentation files as a covariate in the statistical analysis of data; this volume plays a vital role in evaluating hippocampal subfield volumes. Importantly, the reliability and validity of the FreeSurfer hippocampal segmentation protocol have been previously demonstrated (Iglesias et al., 2015; Kahhale et al., 2023).

### Statistical analysis

All statistical analyses were performed using IBM SPSS Statistics for Windows, version 22.0 (IBM Corp., Armonk, NY, United States). We used analysis of variance with *p* < 0.05 to analyze the normal parameters of all clinical data, including age, pain duration, HAMD, HAMA, PSQI, MoCA, and VAS. The Kruskal–Wallis test was used to compare hippocampal subregion volumes and eTIV among the three groups, with significant differences indicated by *p* < 0.001316 (0.05/38 regions; Bonferroni-corrected for multiple comparisons). All intra-group comparisons were Bonferroni corrected, and significant differences were indicated by *p* < 0.05. Additionally, in each group, we used partial correlation analysis to explore the relationship between cognitive function and hippocampal subregion volumes; *p* < 0.001316 indicated significant differences. Age, eTIV, and disease duration were included as covariates.

## Results

### Clinical characteristics

The general clinical characteristics of the subjects in the present study are shown in Table 1. There were no significant differences in age or years of education among the three groups. The HAMD, HAMA, PSQI, and VAS scores were all significantly higher in the FM with MCI and FM without MCI groups than in the HC group (all *p* < 0.001). Furthermore, compared with the HC group, the FM with MCI group had markedly lower MoCA scores (*p* < 0.001).

**Table 1:** Clinical characteristics of all subjects.

	FM with MCI (*n* = 21)	FM without MCI (*n* = 14)	HC (*n* = 35)	*p*^1^ val	*p*^2^ val	*p*^3^ val
	Mean ± SD	Mean ± SD	Mean ± SD			
Age (year)	48.67 ± 13.59	42.21 ± 9.27	44.09 ± 11.85	0.509	0.368	1.000
Sex (male/femle)	0/21	0/14	0/35	–	–	–
Duration of pain (month)	82.10 ± 118.00	54.43 ± 62.20	–	–	–	–
Education year (year)	13.48 ± 4.15	14.89 ± 3.82	12.69 ± 4.99	1.000	1.000	0.406
HAMD	15.62 ± 5.63	15.36 ± 4.72	3.57 ± 1.80	<0.001	0.998	<0.001
HAMA	13.81 ± 6.35	16.36 ± 5.51	3.71 ± 1.98	<0.001	0.520	<0.001
PSQI	9.86 ± 3.45	10.29 ± 3.41	2.77 ± 2.18	<0.001	1.000	<0.001
MoCA	19.57 ± 3.93	29.07 ± 1.14	28.00 ± 1.41	<0.001	<0.001	0.501
VAS	6.33 ± 1.20	5.71 ± 1.20	0.29 ± 0.52	<0.001	0.378	<0.001

Analysis of variance and Bonferroni post hoc tests were used.^1^FM with MCI group vs. HC group; ^2^FM with MCI group vs. FM without MCI group; ^3^FM without MCI group vs. HC group.

FM, fibromyalgia; MCI, mild cognitive impairment; HC, healthy control; SD, standard deviation; HAMD, Hamilton Depression Rating Scale; HAMA, Hamilton Anxiety Rating Scale; MoCA, Montreal Cognitive Assessment; PSQI, Pittsburgh Sleep Quality Index Scale; VAS, visual analog scale.

### Hippocampal subfield volumes

**Figure 2:**
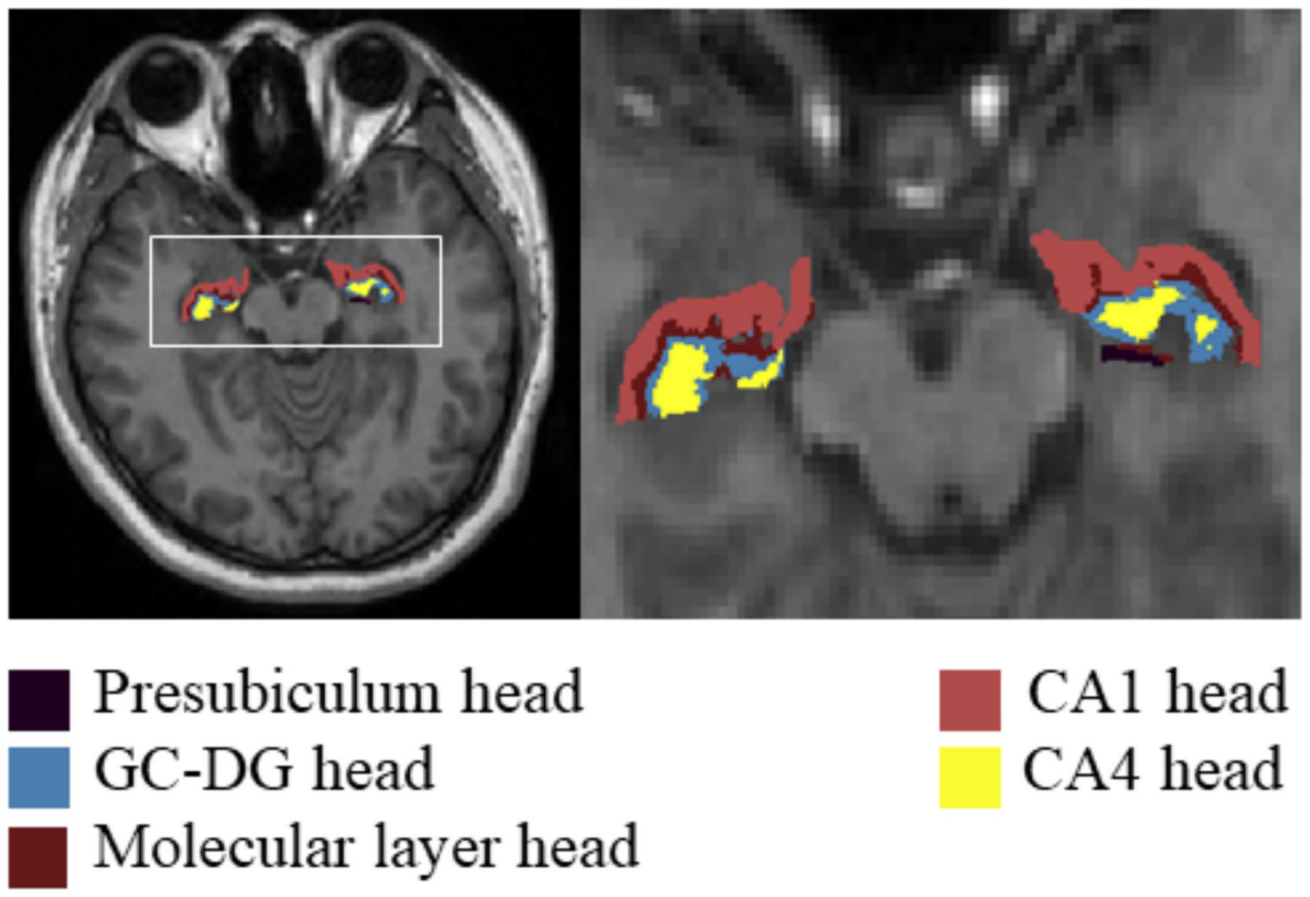
Differences in hippocampal subfield volumes between the three groups in FreeSurfer. CA, cornus ammonis; GC-DG, granule cell layer of the dentate gyrus.

**Figure 3:**
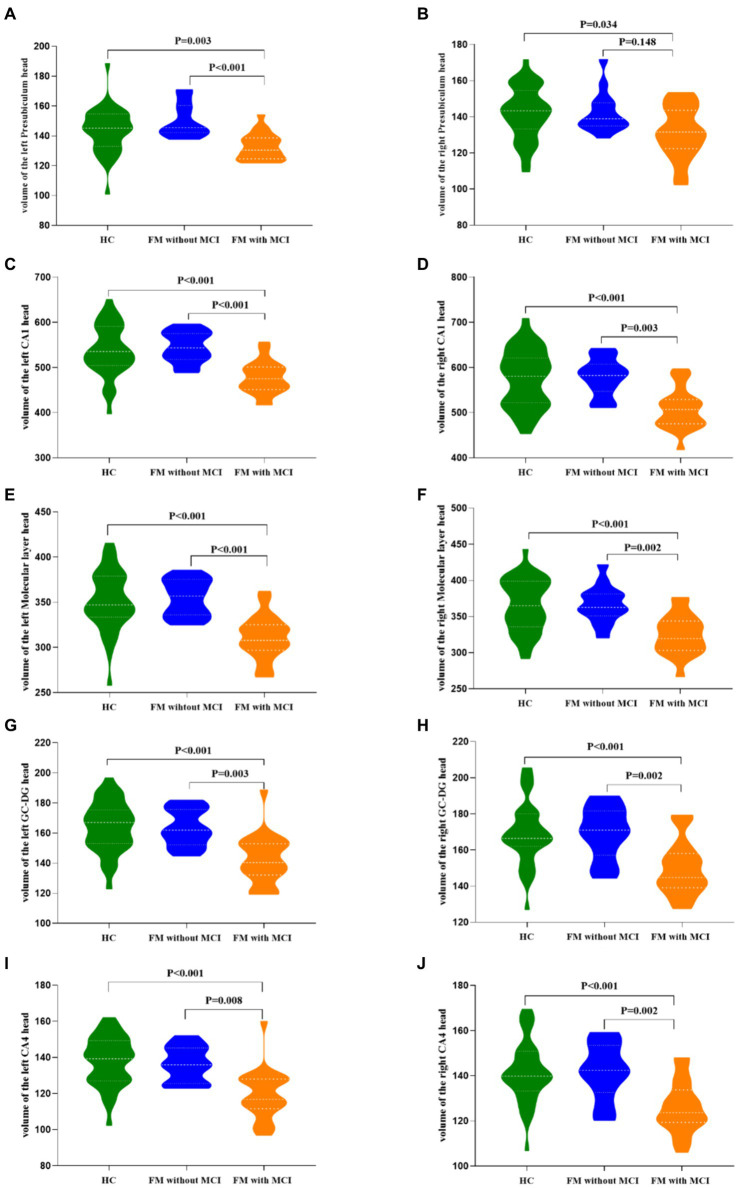
Differences in hippocampal subfield volumes between the three groups. FM, fibromyalgia; MCI, mild cognitive impairment; HC, healthy control; CA, cornu ammonis; GC-DG, granule cell layer of the dentate gyrus.

An apparent difference in eTIV among the three groups was not significant (Table 2). Compared with the HC group, the total bilateral hippocampal volume showed a reducing trend in the FM with MCI group; however, no such change was observed in the FM without MCI group. The volumes of the bilateral CA1 head (left: *p* < 0.001; right: *p* < 0.001), Molecular layer head (left: *p* < 0.001; right: *p* < 0.001), GC-DG head (left: *p* < 0.001; right: *p* < 0.001), and CA4 head (left: *p* < 0.001; right: *p* < 0.001) as well as the left Presubiculum head (*p* = 0.003) were smaller in the FM with MCI group than in the HC group (Table 2, Figures 2, 3). By contrast, the hippocampal subregion volumes in the FM without MCI group were slightly larger than or similar to those in the HC group. Compared with the FM without MCI group, the bilateral CA1 head (left: *p* < 0.001; right: *p* = 0.003), Molecular layer head (left: *p* < 0.001; right: *p* = 0.002), GC-DG head (left: *p* = 0.003; right: *p* = 0.002), and CA4 head (left: *p* = 0.008; right: *p* = 0.002), as well as the left Presubiculum head (*p* < 0.001), showed significantly reduced volumes in the FM with MCI group (Table 2, Figures 2, 3).

**Table 2:** Hippocampal subfield volumes and eTIV in the three groups.

	FM with MCI (*n* = 21)	FM without MCI (*n* = 14)	HC (*n* = 35)	*p*^1^ val	*p*^2^ val	*p*^3^ val	*p*^4^ val
	Mean ± SD	Mean ± SD	Mean ± SD				
Left							
Hippocampal tail	577.53 ± 58.39	622.92 ± 45.55	599.37 ± 73.03	n.s	n.s	n.s	n.s
Subiculum body	255.90 ± 21.60	273.85 ± 23.69	268.86 ± 32.32	n.s	n.s	n.s	n.s
CA1 body	118.79 ± 18.35	132.76 ± 13.94	129.82 ± 18.76	n.s	n.s	n.s	n.s
Subiculum head	183.14 ± 20.29	209.00 ± 21.58	203.25 ± 24.81	n.s	n.s	n.s	n.s
Hippocampal fissure	150.37 ± 31.88	160.67 ± 16.95	150.76 ± 24.49	n.s	n.s	n.s	n.s
Presubiculum head	132.78 ± 8.86	150.25 ± 11.51	143.66 ± 15.16	0.000154	0.003	<0.001	n.s
CA1 head	477.22 ± 36.42	544.97 ± 34.50	536.99 ± 56.79	0.000027	<0.001	<0.001	n.s
Presubiculum body	172.54 ± 15.93	177.69 ± 19.85	174.62 ± 27.63	n.s	n.s	n.s	n.s
Parasubiculum	61.54 ± 5.88	67.29 ± 10.81	66.32 ± 9.85	n.s	n.s	n.s	n.s
Molecular layer head	311.22 ± 24.36	354.90 ± 20.49	351.36 ± 33.07	0.000006	<0.001	<0.001	n.s
Molecular layer body	226.84 ± 22.88	245.95 ± 18.72	239.26 ± 22.15	n.s	n.s	n.s	n.s
GC-DG head	142.33 ± 16.57	163.48 ± 12.44	164.11 ± 16.83	0.000041	<0.001	0.003	n.s
CA3 body	86.60 ± 14.34	93.59 ± 16.20	92.64 ± 16.26	n.s	n.s	n.s	n.s
GC-DG body	137.34 ± 10.82	145.64 ± 9.80	145.70 ± 13.30	n.s	n.s	n.s	n.s
CA4 head	119.22 ± 14.06	136.22 ± 9.82	137.88 ± 13.76	0.000054	<0.001	0.008	n.s
CA4 body	121.33 ± 10.61	129.40 ± 10.32	128.38 ± 11.73	n.s	n.s	n.s	n.s
Fimbria	69.67 ± 12.57	78.03 ± 11.42	77.38 ± 17.54	n.s	n.s	n.s	n.s
CA3 head	112.43 ± 14.97	126.15 ± 14.94	129.71 ± 17.28	n.s	n.s	n.s	n.s
HATA	54.03 ± 5.94	59.37 ± 7.21	57.90 ± 7.00	n.s	n.s	n.s	n.s
Whole hippocampal body	1189.02 ± 95.00	1276.91 ± 75.41	1256.66 ± 110.84	n.s	n.s	n.s	n.s
Whole hippocampal head	1593.92 ± 125.17	1811.62 ± 113.18	1791.17 ± 165.58	0.000012	<0.001	<0.001	n.s
Whole hippocampus	3360.47 ± 223.66	3486.34 ± 837.45	3647.20 ± 293.11	<0.0001	0.001	0.005	n.s
Right							
Hippocampal tail	611.68 ± 62.83	660.33 ± 57.68	627.58 ± 66.16	n.s	n.s	n.s	n.s
Subiculum body	250.53 ± 26.42	262.73 ± 18.25	262.65 ± 26.08	n.s	n.s	n.s	n.s
CA1 body	129.07 ± 22.88	147.61 ± 15.60	140.44 ± 19.46	n.s	n.s	n.s	n.s
Subiculum head	183.77 ± 22.02	208.27 ± 22.03	206.97 ± 28.78	n.s	n.s	n.s	n.s
Hippocampal fissure	150.20 ± 28.63	153.77 ± 21.78	155.42 ± 27.13	n.s	n.s	n.s	n.s
Presubiculum head	131.33 ± 14.54	142.37 ± 11.50	141.93 ± 14.62	n.s	n.s	n.s	n.s
CA1 head	508.04 ± 45.61	578.90 ± 42.10	575.59 ± 64.11	<0.0001	<0.001	0.003	n.s
Presubiculum body	158.36 ± 18.82	156.64 ± 17.89	160.72 ± 23.32	n.s	n.s	n.s	n.s
Parasubiculum	59.23 ± 9.62	62.45 ± 13.64	62.45 ± 8.87	n.s	n.s	n.s	n.s
Molecular layer head	324.64 ± 27.59	366.66 ± 26.18	365.67 ± 36.48	0.000065	<0.001	0.002	n.s
Molecular layer body	235.26 ± 27.91	259.80 ± 17.67	251.95 ± 23.83	n.s	n.s	n.s	n.s
GC- DG head	149.80 ± 15.15	169.51 ± 14.69	169.18 ± 17.44	<0.0001	<0.001	0.002	n.s
CA3 body	97.78 ± 17.30	112.02 ± 14.41	107.50 ± 15.96	n.s	n.s	n.s	n.s
GC-DG body	142.34 ± 16.09	152.94 ± 10.20	151.48 ± 15.68	n.s	n.s	n.s	n.s
CA4 head	125.86 ± 11.48	141.86 ± 12.51	141.49 ± 13.66	0.000089	<0.001	0.002	n.s
CA4 body	127.36 ± 15.98	138.20 ± 10.99	136.17 ± 15.35	n.s	n.s	n.s	n.s
Fimbria	66.87 ± 10.85	76.23 ± 14.00	73.70 ± 10.70	n.s	n.s	n.s	n.s
CA3 head	118.57 ± 14.2722	133.60 ± 16.65	135.41 ± 15.20	n.s	n.s	n.s	n.s
HATA	55.27 ± 6.49	60.58 ± 8.10	60.40 ± 7.84	n.s	n.s	n.s	n.s
Whole hippocampal body	1207.57 ± 121.86	1306.17 ± 74.39	1284.60 ± 112.77	n.s	n.s	n.s	n.s
Whole hippocampal head	1656.51 ± 138.24	1864.20 ± 130.11	1859.08 ± 183.00	0.000097	<0.001	0.002	n.s
Whole hippocampus	3475.77 ± 263.05	3830.70 ± 213.35	3771.26 ± 311.43	0.000620	0.003	0.003	n.s
eTIV	1352563.39 ± 81953.16	1425562.65 ± 82326.70	1405637.92 ± 112064.85	0.066	n.s	n.s	n.s

All volumes are shown as mm^3^.^1^Kruskal–Wallis test at *p* < 0.001316 (0.05/38), comparison among the three groups; ^2,3,4^Mann–Whitney U test for post hoc comparison at *p* < 0.05; ^2^FM with MCI group vs. HC group; ^3^FM with MCI group vs. FM without MCI group; ^4^FM without MCI group vs. HC group.

FM, fibromyalgia; MCI, mild cognitive impairment; HC, healthy control; SD, standard deviation; eTIV, extracted total intracranial volume; CA, cornu ammonis; GC-DG, granule cell layer of the dentate gyrus; HATA, hippocampal amygdala transition area.

### Relationships between cognitive function and hippocampal subfield volumes

**Figure 4:**
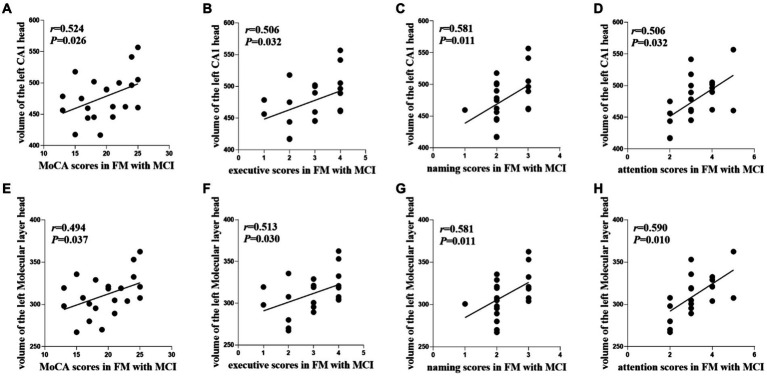
Relationships between cognitive functions and hippocampal subfield volumes in the FM with MCI group. FM, fibromyalgia; MCI, mild cognitive impairment; MoCA, Montreal Cognitive Assessment; CA, cornu ammonis.

In the FM with MCI group, the left CA1 head (*r* = 0.524, *p* = 0.026) and left Molecular layer head (*r* = 0.494, *p* = 0.037) volumes were positively correlated with MoCA scores (Table 3, Figure 4). Moreover, the left CA1 head volume was positively correlated with executive function (*r* = 0.506, *p* = 0.032), naming ability (*r* = 0.581, *p* = 0.011), and attention (*r* = 0.506, *p* = 0.032), whereas atrophy of the left Molecular layer head was related to executive function (*r* = 0.513, *p* = 0.030), naming ability (*r* = 0.581, *p* = 0.011), and attention (*r* = 0.590, *p* = 0.010). There were no significant correlations between hippocampal subregion volumes and any other cognitive functions (i.e., language, abstraction, delayed recall, or orientation). In the HC and FM without MCI groups, hippocampal subfield volumes were not significantly correlated with cognitive function.

**Table 3:** Relationships between cognitive functions and hippocampal subfield volumes in the fibromyalgia with mild cognitive impairment group.

	MoCA		Executive		Naming		Attention	
	r	p	r	p	r	p	r	p
Left								
Presubiculum head	0.411	0.090	0.385	0.114	0.373	0.127	0.339	0.169
CA1 head	0.524	0.026	0.506	0.032	0.581	0.011	0.506	0.032
Molecular layer head	0.494	0.037	0.513	0.030	0.581	0.011	0.590	0.010
GC-DG head	0.256	0.305	0.341	0.166	0.408	0.093	0.646	0.004
CA4 head	0.202	0.422	0.295	0.235	0.334	0.176	0.601	0.008
Right								
CA1 head	0.442	0.067	0.469	0.050	0.570	0.014	0.306	0.216
Molecular layer head	0.413	0.088	0.419	0.083	0.525	0.025	0.278	0.265
GC-DG head	0.243	0.331	0.323	0.192	0.452	0.060	0.249	0.319
CA4 head	0.216	0.389	0.292	0.240	0.433	0.072	0.204	0.417

	Language		Abstraction		Delayed recall		Orientation	
	r	p	r	*p*	r	p	r	p
Left								
Presubiculum head	0.124	0.623	0.364	0.137	0.258	0.302	0.102	0.688
CA1 head	0.114	0.651	0.343	0.163	0.215	0.391	0.178	0.480
Molecular layer head	0.061	0.811	0.268	0.282	0.156	0.538	0.085	0.737
GC-DG head	0.018	0.943	0.101	0.691	−0.171	0.497	−0.295	0.235
CA4 head	−0.030	0.905	0.079	0.755	−0.181	0.472	−0.317	0.200
Right								
CA1 head	0.132	0.600	0.324	0.190	0.143	0.571	0.158	0.531
Molecular layer head	0.260	0.298	0.191	0.447	0.141	0.577	0.182	0.470
GC-DG head	0.049	0.847	0.164	0.515	−0.136	0.590	0.012	0.963
CA4 head	0.035	0.892	0.125	0.622	−0.121	0.632	0.028	0.914

MoCA, Montreal Cognitive Assessment; CA, cornu ammonis; GC-DG, granule cell layer of the dentate gyrus.

## Discussion

In the present study, we segmented hippocampal subregions using FreeSurfer to reveal the volumes of hippocampal subregions in FM patients with or without MCI. We then investigated the relationship between cognitive functions and hippocampal subregion volume alterations in FM patients. We noted the following major findings: (1) In FM patients with MCI, atrophy was observed in the bilateral CA1 head, Molecular layer head, GC-DG head, and CA4 head hippocampal subregions as well as in the left Presubiculum head, (2) Atrophy of the left CA1 head and Molecular layer head was related to executive function, naming ability, and attention in FM patients with MCI, and (3) Hippocampal subregion volumes in FM patients without MCI were slightly larger than or similar to those in the HCs, and were not significantly correlated with cognitive functions in these two groups.

Previous studies have demonstrated that total bilateral hippocampal volume is significantly smaller in FM patients than in HCs (McCrae et al., 2015). In the present study, there was a trend toward a reduction in total hippocampal volume in the FM patients with MCI, similar to previous reports. However, unlike previous studies, we used advanced methods to segment the whole hippocampus into subregions, and adopted a very specialized and detailed functional analysis of hippocampal subregions to explore the relationships between structural changes and cognitive impairments in FM. We identified significant atrophy in many hippocampal subregions of FM patients with MCI, especially in the bilateral CA1 head, Molecular layer head, GC-DG head, and CA4 head, as well as in the left Presubiculum head. By contrast, no atrophy was observed in the hippocampal subregions of FM patients without MCI. Notably, the atrophied hippocampal subregions in the present study differed slightly from those previously reported (Leon-Llamas et al., 2021). However, in the present study, FM patients were divided into two subgroups based on their MoCA scores, whereas most previous studies have examined FM patients as a whole, thus neglecting the effects of cognitive differences on experimental results. This may be one of the reasons for the observed discrepancies. Importantly, our findings suggest that hippocampal subregions may play an important role in the development of cognitive impairment in FM patients.

As noted in the Introduction, the hippocampus is not a single anatomical structure; it is composed of more than a dozen subregions with diverse functions. However, prior investigations have mostly focused on overall hippocampal changes in FM patients, and few studies have examined changes in hippocampal subregions. Furthermore, no studies have explored the correlations between FM-related cognitive impairments and hippocampal subregion volume changes. Therefore, in the present study, FM patients were categorized into two groups based on cognitive function, and the unilateral hippocampus was divided into 19 subregions using FreeSurfer. Compared with earlier studies, we used a more precise segmentation method to segment hippocampal subregions, which helped us to accurately identify small changes associated with FM-related early cognitive decline. It has been reported that FM patients have altered hippocampal metabolites and improved cognitive function after treatment with N-methyl-d-aspartic acid receptor antagonists (Fayed et al., 2019); this earlier finding further supports our hypothesis that alterations in hippocampal microstructure may be involved in the process of cognitive decline in FM patients.

In the current study, significant atrophy was identified in the bilateral CA1-head, Molecular layer head, GC-DG head, and CA4 head, as well as in the left Presubiculum-head, in FM patients with MCI. In several previous studies of hippocampal subregion structure in MCI, different patterns of asymmetric atrophy were noted compared with those of the entire hippocampus (Sarica et al., 2018; Zeng et al., 2021). In the present study, the hippocampus exhibited symmetrical atrophy, which is inconsistent with these previous reports. However, FM patients have multiple, widely distributed pain sites, and our clinical data suggested that pain duration was longer in FM patients with MCI than in those without MCI. Furthermore, previous studies have demonstrated that pain plays a vital role in volume changes of hippocampal subregions (Ezzati et al., 2014; Noorani et al., 2022). For example, Vaculik et al. (2019) investigated the impact of trigeminal neuralgia on hippocampal subregions, and reported that atrophy in selected hippocampal subregions was positively correlated with pain duration. Together, these findings suggest that multiple, widely distributed pain sites and prolonged pain may lead to symmetrical changes in hippocampal atrophy.

Many basic and clinical experiments have confirmed the roles of hippocampal subregions in cognitive decline. A structural MRI study demonstrated a trend toward significantly lower CA1, Presubiculum, ML, and Fimbria volumes in patients with subjective cognitive decline and amnestic MCI (Zhao et al., 2019). Furthermore, in a cohort study with a large sample size, the degree of atrophy in the Presubiculum, Subiculum, and Fimbria was related to poorer cognitive function and a higher risk of dementia, primarily in the form of impaired executive functioning (Evans et al., 2018). Some of our findings are consistent with these previous studies. From an anatomical perspective, we investigated the hippocampal subregions associated with cognitive impairment; we included the CA1 and ML, which are located on the outer side of the hippocampus. Previous anatomical and physiological studies have indicated that the hippocampal information transmission circuit starts in the DG before passing through the projection pathway between the CA1 and subiculum; the CA1 is therefore involved in pathways that regulate the activity of hippocampal circuits and learning/memory (Xu et al., 2016; Ginsberg et al., 2019). In a prior study, patients with FM were reported to have poorer working memory compared to those without FM (Kratz et al., 2020). In the present investigation, we identified significant atrophy of the left CA1 head in FM patients with MCI; this atrophy was correlated with executive function and attention. Notably, the ML is located between the subiculum and the fissure. A previous report revealed that cognitive function in patients with early Alzheimer’s disease or MCI is significantly correlated with synaptic number in the ML (Scheff et al., 2006). A reduction in synaptic number in the hippocampal ML may influence the exchange of information between pyramidal cells and interneurons, which in turn may affect connectivity between subregions, and might ultimately lead to cognitive impairment with prominent memory decline. Similarly, we identified a correlation between ML atrophy and cognitive function in the present study. Together, these findings suggest that cognitive impairment in FM patients may involve multiple hippocampal subregions.

In the current study, we observed the disease duration was significantly longer in the FM patients with MCI than in those without MCI, and hippocampal subregion volumes were significantly smaller in the FM patients with MCI than in the HCs, indicating that hippocampal subregion atrophy may precede the onset of cognitive decline. Although FM patients without MCI had no cognitive decline, our experimental results revealed that their hippocampal subregion volumes were slightly larger or similar to those of the HCs. We therefore speculate that a compensatory mechanism may exist in the hippocampal subregion structures of FM patients prior to the onset of MCI symptoms; there may also be a complex mechanism of functional regulation in these early cognitive function changes. To address these issues, extensive future research is needed.

There are several limitations in the current study. First, FM is a gendered pathology, with a 12:1 prevalence ratio in women to men (Marschall et al., 2011). Female FM patients reportedly have different pathological characteristics than male patients, such as more significant sleep disturbances, more frequent fatigue, and pain in multiple areas (Yunus, 2001). Although the mechanisms of sex differences in FM incidence have not yet been fully elucidated, their possible causes may be explained by the interplay of biological, physiological, and social experiential factors between sexes (Arout et al., 2018). Research into FM should therefore consider evaluating the differences in these characteristics between sexes. However, all subjects in the present study were women, meaning that our results cannot be generalized to male FM patients. Second, our study was based on cross-sectional data, and any longitudinal changes in hippocampal volume were unable to be assessed in the FM patients. Future studies therefore need to incorporate longitudinal follow-up data, to better explain the changes in cognitive function in FM patients. In addition, research combining cross-sectional and longitudinal follow-up data may help to determine whether specific hippocampal subregion atrophy can predict cognitive function decline, and thus identify relevant targets for prevention and/or treatment. For example, parietal insular repetitive transcranial magnetic stimulation in patients with Alzheimer’s disease leads to short-term improvements in cognitive function (Wei et al., 2022). Future research should therefore also consider repetitive transcranial magnetic stimulation as a treatment for FM patients, to observe whether structural reconstruction of hippocampal subregions occurs, thereby improving cognitive function.

## Conclusion

The present study provides evidence of hippocampal subregion atrophy in FM patients with MCI, as well as associations between specific hippocampal subregions and cognitive decline. These specific associations will help us to identify early cognitive decline in FM and develop interventions. They will also aid our understanding of the potential biological mechanisms underlying FM-related cognitive deficits and hippocampal subregion volume alterations.

## Data availability statement

The original contributions presented in the study are included in the article/supplementary material, further inquiries can be directed to the corresponding authors.

## Ethics statement

The studies involving humans were approved by the Research Ethics Committee of the Affiliated Hospital of the Nanjing University of Chinese Medicine (2021NL-193-02). The studies were conducted in accordance with the local legislation and institutional requirements. The participants provided their written informed consent to participate in this study.

## Author contributions

YL: Conceptualization, Data curation, Formal analysis, Investigation, Methodology, Visualization, Writing – original draft, Writing – review & editing. XX: Data curation, Investigation, Writing – original draft, Writing – review & editing. YW: Data curation, Investigation, Methodology, Writing – original draft, Writing – review & editing. JX: Writing – review & editing. ZG: Data curation, Investigation, Software, Writing – review & editing. XF: Investigation, Methodology, Software, Supervision, Writing – review & editing. TX: Data curation, Investigation, Writing – review & editing. NZ: Data curation, Investigation, Writing – review & editing. DL: Funding acquisition, Project administration, Resources, Writing – review & editing. TW: Funding acquisition, Project administration, Resources, Writing – review & editing.

## Footnotes

^1^ https://surfer.nmr.mgh.harvard.edu/
